# Queen reproductive tract secretions enhance sperm motility in ants

**DOI:** 10.1098/rsbl.2016.0722

**Published:** 2016-11

**Authors:** Joanito Liberti, Boris Baer, Jacobus J. Boomsma

**Affiliations:** 1 Centre for Social Evolution, Department of Biology, University of Copenhagen, Universitetsparken 15, 2100, Copenhagen, Denmark; 2 Centre for Integrative Bee Research (CIBER), The University of Western Australia, Bayliss Building M316, Crawley, Western Australia 6009, Australia

**Keywords:** social insects, sexual selection, sperm competition, sperm motility, cryptic female choice

## Abstract

Queens of *Acromyrmex* leaf-cutting ants store sperm of multiple males after a single mating flight, and never remate even though they may live for decades and lay tens of thousands of eggs. Sperm of different males are initially transferred to the bursa copulatrix and compete for access to the long-term storage organ of queens, but the factors determining storage success or failure have never been studied. We used *in vitro* experiments to show that reproductive tract secretions of *Acromyrmex echinatior* queens increase sperm swimming performance by at least 50% without discriminating between sperm of brothers and unrelated males. Indiscriminate female-induced sperm chemokinesis makes the likelihood of storage directly dependent on initial sperm viability and thus provides a simple mechanism to secure maximal possible reproductive success of queens, provided that initial sperm motility is an accurate predictor of viability during later egg fertilization.

## 1. Introduction

In promiscuous mating systems, sperm compete for direct egg fertilization or access to storage sites, whereas females may bias the outcome by chemically modulating the direction (chemotaxis) or speed (chemokinesis) of sperm motility [1,2]. Faster-swimming sperm are usually more successful in fertilizing eggs [3–5], but very little is known about the actual processes involved and the magnitude of effects exerted by female secretions. Quantifying such effects is difficult because interindividual variation in duration of sperm storage and time towards female re-mating is normally high [6,7]. The social Hymenoptera (ants, bees and wasps) offer interesting exceptions to this rule, because mate choice and insemination are restricted to a single brief time window early in adult life, during which virgin queens store all the sperm they will ever obtain during their life.

Exclusive single queen mating is ancestral in all ants, bees and wasps that evolved morphologically distinct queen and worker castes [8,9], but polyandry (the storage of multiple ejaculates) evolved in several evolutionarily derived lineages [10]. In such clades, ejaculates compete for access to the queen's sperm storage organ (the spermatheca), from where sperm will be used to fertilize eggs for up to several decades [11]. Because re-mating later in life is impossible, a queen's lifetime reproductive fitness will depend on the quantity and viability of the sperm stored after early life insemination [12]. This should imply strong selection for storing only viable sperm until the maximal storage capacity is reached.

*Acromyrmex* leaf-cutting ants are highly suitable to investigate whether virgin queens have evolved mechanisms to preferentially store sperm of the highest possible quality because: (i) all queens are inseminated by multiple males [13,14], (ii) queens have a large fluid-filled bursa copulatrix where ejaculates are deposited before a fraction of sperm can enter the smaller spermatheca by active motility [13,15] and (iii) the time span between insemination and final storage is only a few hours [13]. We used *Acromyrmex echinatior* to investigate whether queen reproductive tract secretions affect sperm motility such that faster sperm are more likely to become stored and whether any such effects are universal or discriminate against related sperm, as inbreeding in haplodiploid insects can incur fitness costs by increasing the probability of diploid larvae developing into sterile males [16,17].

## 2. Material and methods

Colonies of *A. echinatior* were excavated in Gamboa, Panama from 2001 to 2014 and reared in Copenhagen at 25°C and relative humidity 60–70%. Winged reproductives were collected shortly before each trial and checked for sexual maturity during dissection [18] with watchmaker forceps in Hayes saline (see the electronic supplementary material for details).

Accessory testes of 16 males were punctured to collect subsamples of outflowing sperm with a pipette tip previously loaded with 3 µl Hayes saline containing 375 µM of SYTO 13 (Molecular Probes) fluorescent dye (see the electronic supplementary material for details). Mixtures were gently pipetted into a counting chamber (SC-20-01-04-B, Leja), after which spermatozoa were observed with a spinning-disc confocal microscope (Revolution XD, Andor) at 20× magnification. The fluorescent dye was excited with a 488 nm laser and motility recorded at 30 frames per second with an Andor iXon DU-897-BV EMCCD camera. For each male, we obtained two 5 s recordings, between which we changed the field of vision within the same counting chamber, expecting that sperm motility parameters should remain similar unless measurements were affected by technical noise. We analysed recordings with the computer-assisted sperm analyser (CASA) plugin [19] for ImageJ (http://imagej.nih.gov/ij/) and assessed measurement repeatability with the R package rptR, method REML [20,21].

**Figure 1.**
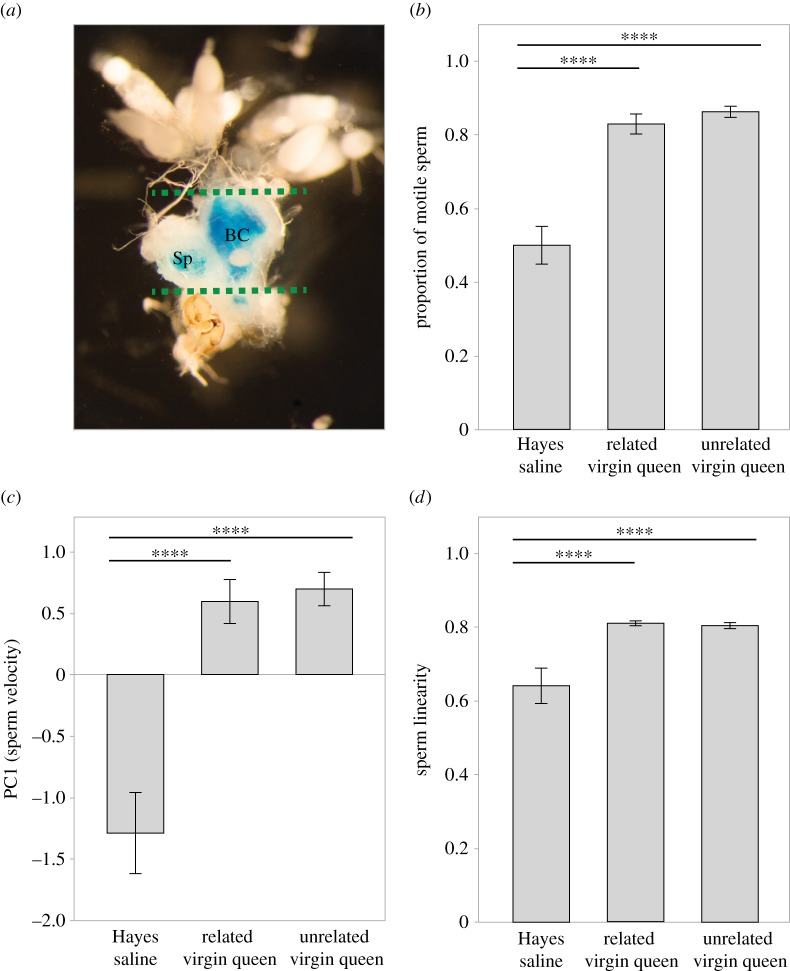
Female (queen) reproductive fluid (*a*) in the leaf-cutting ant *A. echinatior* enhances the proportion of motile sperm (*b*), sperm velocity (*c*) and the linearity of sperm movement (*d*) relative to Hayes saline controls, independently of whether virgin queens are siblings or unrelated. Image (*a*) shows a representative female reproductive tract after dissection. Dotted lines indicate where the bursa copulatrix (BC) and spermatheca (Sp) were separated from the rest of the reproductive tract when used for experiments. To visualize the two sperm storage organs whose fluids were used in the experiments, this particular virgin queen was artificially inseminated with blue dye prior to dissection. Bars are mean ± s.e. and horizontal lines specify significance of differences (****p < 0.0001).

Using the same methods, we obtained 5 s recordings of spermatozoa from 10 individual males (two replicate experiments) while swimming in: (i) reproductive tract fluid from a virgin queen collected from the same colony as the focal male, (ii) virgin queen fluid from an unrelated colony and (iii) Hayes saline as a control. To obtain female fluid, we dissected the bursa copulatrix and spermatheca of virgin queens (figure 1*a*), gently punctured these organs in 3 µl Hayes in a 0.2 ml PCR tube, centrifuged at 17 000***g*** for 3 min, and transferred 1.5 µl supernatant (or Hayes only) into 2 µl Hayes containing SYTO 13 (375 µM final concentration) before using 3 µl aliquots as test fluids.

Because CASA yields intercorrelated measures of sperm velocity, we performed a principal component analysis (JMP v. 12) incorporating curvilinear velocity (VCL), velocity on the average path (VAP) and straight-line velocity (VSL) and used the first principal component (PC1) as a proxy of overall sperm velocity. PC1, the proportion of motile sperm and sperm linearity (LIN) were subsequently used as dependent variables in linear mixed-effects models fitted by restricted maximum likelihood (see the electronic supplementary material for details).

## 3. Results

All measurements of sperm motility within the same counting chamber were highly repeatable, which confirmed that our methods were reliable (proportion of motile sperm: *R* = 0.89, s.e. = 0.05, *p* < 0.0001; VCL: *R* = 0.77, s.e. = 0.10, *p* < 0.0001; VAP: *R* = 0.85, s.e. = 0.10, *p* < 0.0001; VSL: *R* = 0.85, s.e. = 0.07, *p* < 0.0001; LIN: *R* = 0.54, s.e. = 0.18, *p* = 0.013).

The proportion of motile sperm increased by almost 70% (figure 1*b*) when spermatozoa were exposed to queen reproductive fluid compared with Hayes saline (*F*_1,104_ = 69.55, *p* < 0.0001), but reproductive fluids from related and unrelated females had similarly enhancing effects (*F*_1,104_ = 0.48, *p* = 0.49). Sperm velocity (PC1; figure 1*c*) increased by *ca* 50% in queen fluids compared with the Hayes controls (*F*_1,104_ = 49.09, *p* < 0.0001), similar to the three original variables that loaded PC1 (49.4% for VCL, 52.2% for VAP, 57.2% for VSL) and once more without segregation between related and unrelated female fluids (*F*_1,104_ = 0.10, *p* = 0.75). Sperm movement (figure 1*d*) was consistently more linear (25.8% increase) when swimming in female fluid compared with Hayes saline (*F*_1,104_ = 23.35, *p* < 0.0001), with no difference between related and unrelated female reproductive fluids (*F*_1,104_ = 0.01, *p* = 0.91).

**Figure 2.**
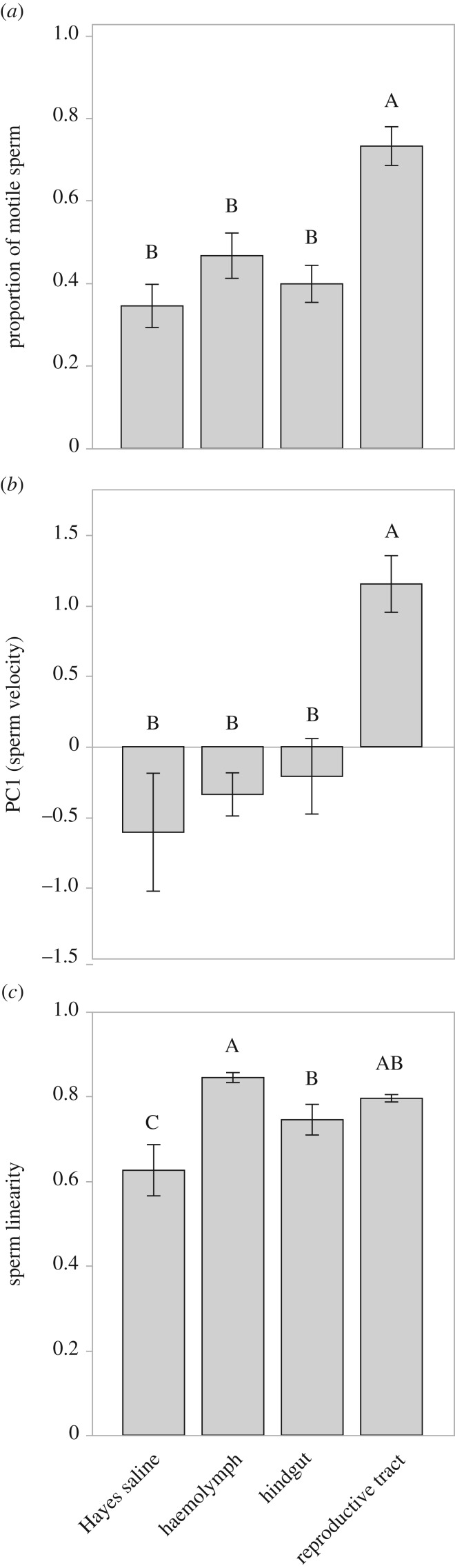
Queen reproductive tract fluid enhances the proportion of motile spermatozoa (*a*) and sperm velocity (*b*) compared with haemolymph, hindgut secretion and Hayes saline, but reproductive tract fluid, haemolymph and hindgut secretion have similar positive effects on sperm linearity (*c*). Bars show mean ± s.e. and levels marked with different letters were significantly different (Student's *t*-tests).

To exclude the possibility that proteins or other compounds from non-reproductive tissues could have similar effects, we ran an extra series of controls repeating the experiment with haemolymph and hindgut fluid. The effects of these extra controls were identical to Hayes saline for the proportion of motile sperm and sperm velocity, but similar to reproductive tract fluid for sperm linearity (figure 2), suggesting that only the effects on sperm motility and velocity are induced by compounds specific to the female reproductive tract.

## 4. Discussion

Our results indicate that the fluid contained in the reproductive tract of *A. echinatior* queens activates sperm and increases swimming performance by at least 50% for both unrelated and related (brother) sperm. These *in vitro* effects may well be underestimates of what happens during natural insemination and sperm storage because our experiments used substantial dilutions of female reproductive tract fluids. The results are consistent with these female secretions having evolved mechanisms analogous to the mammalian sperm hyperactivation process [22] to ensure that only the most viable sperm become stored in the spermatheca, where they will stay viable for a potential life span of up to two decades. Our finding that female reproductive tract fluid indiscriminately affects related and unrelated sperm does not refute that inbreeding can incur fitness costs, as matched matings at the sex determining locus are known to impose genetic load on *Acromyrmex* colonies in the form of diploid males [16]. Rather, it suggests that chemokinesis and self–non-self recognition cannot be combined, or that the likelihood of small effective population size and inbreeding by chance is sufficiently low to preclude selection for costly discrimination. Whether kin recognition would occur with undiluted secretions remains to be seen, but such an artefactual explanation seems unlikely as other effects of reproductive fluids are rather insensitive to dilution (this study and [15,23]).

Our results are consistent with the exceptional selection forces on sperm competition and storage that we outlined in the Introduction as being unique for the social Hymenoptera. Even in evolutionarily derived lineages where multiple insemination is the norm, ant queens accumulate all the sperm they will ever have in a single mating flight just after reaching sexual maturity. Earlier studies showed that male seminal fluid of *Acromyrmex* and *Atta* leaf-cutting ants incapacitate other male's sperm after insemination [15], and that spermathecal secretions neutralize this negative effect in *Atta* where sperm is deposited almost immediately in the spermatheca without protracted pre-storage in the bursa copulatrix [23,24]. However, *Acromyrmex* sperm need to actively swim towards final storage [13]. This suggests that the two leaf-cutting ant genera have fundamentally different mechanisms of female control over sperm incapacitation among competing males, with *Atta* queens apparently using mass elimination of sperm competition upon final storage and *Acromyrmex* using individual sperm chemokinesis. Our *in vitro* experimental results suggest that *Acromyrmex* queens fill their spermatheca gradually while prioritizing the most viable sperm present in a larger pool of candidate sperm in the fluid-filled bursa copulatrix [13]. As the *Atta* sperm storage mechanism is truly exceptional, we expect that our present findings for *Acromyrmex* are more representative for ants in general.

Our study shows that the unusual characteristics of ant mating systems based on lifetime commitment of sexual partners provide interesting opportunities to test aspects of sperm competition and female manipulation of sperm storage, which cannot be experimentally manipulated with the same feasibility in solitary insects where promiscuous re-mating across the female life span is the norm.

## Supplementary Material

Extended methods and results

## Supplementary Material

Datasets supporting this article

## Data accessibility

The datasets supporting this article have been uploaded as part of the electronic supplementary material.
