# Supplementary material for: Queen reproductive tract secretions enhance sperm motility in ants
Source: Biol Lett. 2016 Nov;12(11):20160722. doi: 10.1098/rsbl.2016.0722 (PMC5134051; doi:10.1098/rsbl.2016.0722)
Supplement: Extended methods and results [file rsbl20160722supp1.docx]

**Electronic Supplementary Materials**

**Queen reproductive tract secretions enhance sperm motility in ants**

Joanito Liberti^1^, Boris Baer^2^ and Jacobus J. Boomsma^1^

**Affiliations:**

^1^Centre for Social Evolution, Department of Biology, University of Copenhagen, Universitetsparken 15, DK-2100, Copenhagen, Denmark

^2^Centre for Integrative Bee Research (CIBER), Bayliss Building M316, The University of Western Australia, Crawley, WA 6009, Australia

**Corresponding authors:**

Joanito Liberti ([joanito.liberti@bio.ku.dk](mailto:Joanito.liberti@bio.ku.dk)); Jacobus J. Boomsma ([jjboomsma@bio.ku.dk](mailto:jjboomsma@bio.ku.dk))

*Computer Assisted Sperm Analyzer (CASA) system and microscopy procedures*

Commercial CASA systems have been widely used for automated and unbiased analyses of sperm motility in humans and some other animal taxa [1-4]. In recent years, open-source software has allowed for greater control of parameter settings and enhanced repeatability of data obtained [5]. However, some technical challenges remain when sperm morphology compromises software-based discrimination and subsequent tracking of sperm heads from phase contrast images. This is why very few studies have as yet examined insect sperm motility (reviewed in [6]; see also [7]) and no study has, to our knowledge, used this approach to simultaneously estimate the key variables that are commonly measured in human fertility studies in an experimentally controlled and reproducible manner.

*Acromyrmex echinatior* spermatozoa have elongated heads that are indistinguishable from sperm flagella under common light-microscopy. We therefore used a cell-permeant nucleic acid stain (SYTO 13, Molecular Probes), which was found to consistently enhance contrast between sperm heads and flagella and to stain cells within less than a minute from application when used at a concentration of 375 µM (see below). Pilot observations indicated that the use of this fluorochrome did not impair *A. echinatior* sperm motility, but we nevertheless designed our study such that any effect of SYTO 13 on sperm motility parameters would have affected sperm cells equally across treatment groups.

The use of a fluorochrome requires a video recording system that is sensitive enough to capture the fluorescent signals at a high frame rate. We therefore used a spinning-disk confocal microscope (Revolution XD, Andor) equipped with a Yokogawa CSU-X1 Spinning Disk Unit, an Andor iXon DU-897-BV EMCCD camera, and a 20X dry objective when exciting the fluorochromes with a 488 nm laser. The EM gain of the camera was set to 60 and the exposure time to 32 ms, which allowed us to obtain well-defined videos of sperm movements at 30 frames per second (fps; figure S2).

**Figure S2** Representative frame from a video recording of *A. echinatior* spermatozoa.
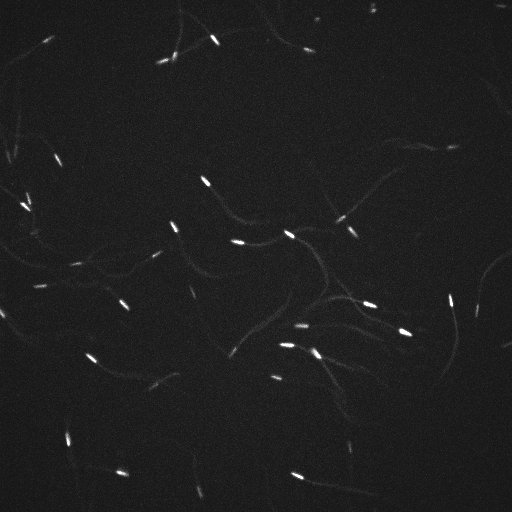


Males were dissected with watchmaker forceps in Hayes saline (9 g NaCl, 0.2 g CaCl_2_, 0.2 g KCl and 0.1 g NaHCO_3_ in 1000 ml H_2_O, adjusted to pH 8.7 and sterilized by filtration through a 0.22 µm syringe-filter, Membrane Solutions). Abdomens were gently opened, the accessory testes exposed and punctured, and subsamples of outflowing sperm collected with a pipette tip previously loaded with either pure Hayes saline or Hayes-diluted reproductive secretion of a related or unrelated female, each containing 375 µM of SYTO 13 (Molecular Probes) fluorescent dye. Hayes does not contain any carbohydrates, fatty- or amino acids and therefore makes it possible to study the effects of queen secretions on sperm motility in an osmotically regulated environment without external energy sources [8].

To add the dye, we first created a working solution of 500 µM SYTO 13 by diluting the SYTO 13 stock solution (5 mM in DMSO) by a factor 10 with Hayes saline. Then, 2 µl of the SYTO 13 working solution were added to 1.5 µl of the test fluids to achieve a final concentration of 375 µM SYTO 13. Three µl of these sperm-containing solutions were then gently pipetted into a counting chamber (SC-20-01-04-B, Leja) and visualized and recorded with the Andor Revolution XD spinning-disk confocal microscope as explained above.

*Repeatability of sperm motility measurements within males*

To check the repeatability of our measurements we recorded sperm movements of individual males in two subsequent 5 s recordings between which we changed the field of vision within the same counting chamber. We measured ejaculates from a total of 16 males collected from 11 different colonies (table S1). Video recordings were analyzed with the CASA plugin (see below for parameter details) for ImageJ [5], tracking an average of 75 (± 45.6 SD) spermatozoa per video. Measurement repeatability was subsequently assessed with the R package rptR, method REML [9, 10].

**Table S1** Collection of males sampled for the repeatability test of sperm motility measurements within males, with IDs of natal colonies and year of colony collection in Panama.

| **Trial** | **Male donor colony** | **Year of collection** |
| --- | --- | --- |
| 1 | Ae420A | 2009 |
| 2 | Ae226B | 2003 |
| 3 | Ae168 | 2002 |
| 4 | Ae372 | 2008 |
| 5 | Ae322 | 2006 |
| 6 | Ae342 | 2007 |
| 7 | Ae506 | 2011 |
| 8 | Ae704 | 2014 |
| 9 | Ae507 | 2011 |
| 10 | Ae332 | 2007 |
| 11 | Ae376 | 2008 |
| 12 | Ae704 | 2014 |
| 13 | Ae507 | 2011 |
| 14 | Ae342 | 2007 |
| 15 | Ae506 | 2011 |
| 16 | Ae376 | 2008 |

*Sperm motility when exposed to queen reproductive secretions*

In order to quantify the effects of reproductive fluids of related and unrelated females on sperm motility variables, spermatozoa obtained from individual males were recorded while swimming in the reproductive fluid of virgin queens collected either from the same colony as the focal male or from an unrelated colony, or in Hayes saline as a control.

Virgin queens were dissected in Hayes. Their reproductive tract was separated from the gaster (rear end of the abdomen) by pulling the last abdominal sclerites, after which the *bursa copulatrix* and spermatheca could be isolated by removing the lateral oviducts, the mussel organ and any fat body tissue (figure 1a), and placed in 3 µl Hayes in a 0.2 ml PCR tube. After gently puncturing the female tissues, we centrifuged at 17,000 g for 3 min after which 1.5 µl supernatant (or Hayes only) was transferred into 2 µl SYTO 13 working solution, briefly vortexed and spun down with a mini-centrifuge. Three µl of the final solutions were then used as test fluids in counting chambers (SC-20-01-04-B, Leja) and sperm movements in these fluids were recorded as previously explained.

We used a total of 12 ant colonies and never employed a colony more than once as male donor or to obtain female reproductive fluid from related and unrelated virgin queens (table S2), and we randomized treatment loading between trials. At the time of sampling colony Ae704 did not provide males, so this colony was only used to obtain unrelated virgin queens. We did 10 trials testing the spermatozoa of the same focal male across the two female fluid treatments and Hayes-only controls in separate counting chambers within the same Leja slide. Two minutes after loading the slide, we recorded two series of 5 s videos moving sequentially across slide chambers. The entire experiment was replicated once using the same colony combinations for both male and female donors involving 10 additional trials. As before, video recordings were analyzed with the CASA plugin for ImageJ, tracking an average of 47.8 (± 34.1 SD) spermatozoa per video.

**Table S2** Combinations of colonies used as male and female donors for the assessment of sperm motility when exposed to reproductive queen secretions. The whole series of ten trials was done twice using identical colony combinations.

| **Trial** | **Male colony** | **Related virgin queen colony** | **Unrelated virgin queen colony** |
| --- | --- | --- | --- |
| 1 | Ae332 | Ae332 | Ae361 |
| 2 | Ae361 | Ae361 | Ae506 |
| 3 | Ae506 | Ae506 | Ae420A |
| 4 | Ae420A | Ae420A | Ae507 |
| 5 | Ae507 | Ae507 | Ae376 |
| 6 | Ae376 | Ae376 | Ae704 |
| 7 | Ae372 | Ae372 | Ae342 |
| 8 | Ae342 | Ae342 | Ae226B |
| 9 | Ae226B | Ae226B | Ae322 |
| 10 | Ae322 | Ae322 | Ae168 |

*Sperm motility when exposed to additional queen body secretions*

In order to assess to what extent the effects on sperm motility parameters are specifically induced by components of the queen reproductive tract fluid, we performed a follow up experiment including a series of additional controls where the same male spermatozoa were tested against reproductive tract fluid, hemolymph and hindgut tissue secretions of the same virgin queens, or Hayes saline only. One µl of hemolymph was obtained by mechanical compression of the thorax between thumb and index finger after removal of the abdomen and head, so that droplets of hemolymph could be collected in a glass capillary from these openings [11] and placed in 3 µl of Hayes in a 0.2 ml PCR tube. The reproductive tract and hindgut were obtained by dissection of the abdomen and placed in 3 µl Hayes in separate 0.2 ml PCR tubes. All tubes were centrifuged as explained above, after which the supernatants were placed in 2 µl Hayes containing SYTO 13 and 3 µl of these fluids were then tested as before. We performed two identical series of 8 trials with the colony combinations shown in Table S3. Video recordings were analyzed with the CASA plugin for ImageJ, tracking an average of 64.8 (± 45.3 SD) spermatozoa per video.

**Table S3** Combinations of colonies used as male and female donors for the assessment of sperm motility when exposed to a series of queen body secretions. The whole series of eight trials was done twice using identical colony combinations. Colonies Ae150A and Ae150B were collected in 2001, Ae263 in 2004, Ae356 in 2008 and Ae480 in 2010. See table S1 for the year of collection of the remaining colonies.

| **Trial** | **Male colony** | **Virgin queen colony** |
| --- | --- | --- |
| 1 | Ae226B | Ae332 |
| 2 | Ae332 | Ae480 |
| 3 | Ae480 | Ae263 |
| 4 | Ae263 | Ae356 |
| 5 | Ae356 | Ae150A |
| 6 | Ae150A | Ae150B |
| 7 | Ae150B | Ae506 |
| 8 | Ae506 | Ae226B |

*Determination of CASA parameters and analyses of video recordings*

Video recordings were analyzed with the CASA plugin [5] for ImageJ (<http://imagej.nih.gov/ij/>). The parameter settings during analysis are dependent on species-specific sperm characteristics and the microscopy system, and were determined experimentally. We used ImageJ to measure sperm head areas and subsequently set the minimum (a) and maximum (b) sizes of the sperm heads to 20 and 250 pixels, respectively. The minimum track length (c; number of frames through which a sperm had to keep moving to be considered motile) was set to 30 (corresponding to 1 s of video recording). The maximum sperm velocity between two subsequent frames (d) was set to 12 pixels/frame unit. The minimum straight-line velocity (e; VSL), minimum velocity on the average path (f; VAP) and minimum curvilinear velocity (g; VCL) for sperm to be considered motile were set to 3, 10 and 10 µm/s, respectively. The maximum percentage of path with zero VAP (i) and low VAP (j) were set to 1 and 10, respectively. Low VAP speed 2 (k) and low VCL speed (l) were both set to 10 µm/s. The frame rate (q) was set to 30 fps and the microns per 1000 pixels parameter (r) was set to 683.3728 µm after taking a photo of a calibrated ruler under the 20X objective used for the video recordings and measuring this distance with ImageJ. All remaining parameters remained at their default setting.

The following macro was used for the automated ImageJ analyses of our video recordings:

run("Set Scale...", "distance=0 known=0 pixel=1 unit=pixel");

//run("Brightness/Contrast...");

setMinAndMax(20, 1500);

call("ij.ImagePlus.setDefault16bitRange", 16);

run("8-bit");

setAutoThreshold("Default dark");

//run("Threshold...");

setThreshold(40, 255);

setOption("BlackBackground", false);

run("Convert to Mask", "method=Default background=Dark");

run("CASA ", "a,=20 b,=250 c,=30 d,=12 e,=3 f,=10 g,=10 h,=5 i,=1 j,=10 k,=10 l,=10 m,=80 n,=80 o,=50 p,=60 q,=30 r,=683.3728 s,=0 t,=1 u,=1”);

When slight differences in the recorded background light intensity required so, the threshold (the parameter that informs the software about which signals represent spermatozoa and background) was manually re-adjusted to improve resolution.

We obtained the following sperm motility variables, which are described in [5]: Proportion of motile sperm: proportion of tracked sperm identified by the plugin as exhibiting motility during the 5 s period of analysis; Curvilinear velocity (VCL): the total point to point distance traveled by the sperm over the time period analyzed, averaged to a per second value; Velocity average path (VAP): velocity over an average path generated by a roaming average of sperm position from one-sixth of the video's frame rate, such that each point is generated by averaging the coordinates of a set number of locations on the VCL path; Velocity straight-line (VSL): velocity measured using the first point and the average path and the point reached that is furthest from this origin during the measured time period. Linearity (LIN) was the VSL/VAP ratio, which describes path curvature. In videos where all sperm cells were found to be non-motile (7 out of 120; all in the Hayes control treatment group) we considered all values to be zero because these ejaculates were motile in the female fluid treatments within the same trials, so lack of motility in the control group was biologically relevant and not merely technical noise. We nevertheless repeated statistical analyses after using the default of not assigning any value to velocity and linearity variables and thus excluding these data points. Statistical results did not differ when we excluded these recordings, so in the main text we report the analyses in which these values were assigned to be zero.

These five variables that we obtained in this manner were all correlated (table S4) and thus unsuitable for direct use in statistical analyses, Similarly to previously published work [4], we largely avoided this multicollinearity problem by using a Principal Component Analysis (PCA) in JMP v. 12, which reduced all three velocity measures to a single principal component with eigenvalue 2.9319 (figure S3) so as to end up with three biologically relevant dependent variables, describing the proportion of motile spermatozoa, their speed and the type of movement they performed, which were used in subsequent analyses.

**Table S4** Pearson correlations between all combinations of variables used in the experiments testing sperm motility in queen secretions.

|  | **VCL** | **VAP** | **VSL** | **LIN** | **Number of tracked sperm** |
| --- | --- | --- | --- | --- | --- |
| Proportion of motile sperm | 0.71 | 0.77 | 0.77 | 0.70 | 0.27 |
| VCL |  | 0.97 | 0.94 | 0.72 | 0.26 |
| VAP |  |  | 0.98 | 0.73 | 0.23 |
| VSL |  |  |  | 0.75 | 0.16 |
| LIN |  |  |  |  | 0.15 |

**Figure S3** Principal Component Analysis of the three sperm velocity measures (VCL, VAP and VSL) in the experiments testing sperm motility in queen secretions.

*Statistical analyses*

Sperm motility data were analyzed using JMP v. 12. We performed separate linear mixed-effects models fitted by restricted maximum likelihood (REML) for PC1, the proportion of motile sperm and sperm linearity (LIN). The two series of ten trials (referred to as “experimental replicate” in tables S5 and S6) and the ten trials within each series (referred to as “trial” in tables S5 and S6) were treated as random effects and treatment, time point (the two series of video recordings in each trial) and their interaction as fixed effects. Detailed results for each dependent variable (including additional analyses for the three variables that loaded PC1) are presented in table S5 and table S6.

**Table S5** Results of linear mixed-effects models fitted by restricted maximum likelihood in the experiments testing sperm motility in queen reproductive tract secretions, including results for the three velocity variables (VCL, VAP and VSL) that loaded PC1, and the percentage of variance explained by random effects. DF = degrees of freedom; DFDen = denominator degrees of freedom. Significant results are presented in bold.

| **Dependent variable** | **Fixed effects** | **Contrast** | **DF** | **DFDen** | **F Ratio** | **p-value** |
| --- | --- | --- | --- | --- | --- | --- |
| Proportion of motile sperm | Treatment |  | 2 | 104 | 35.02 | **<.0001** |
|  |  | Hayes vs related virgin queen | 1 | 104 | 47.26 | **<.0001** |
|  |  | Hayes vs unrelated virgin queen | 1 | 104 | 57.31 | **<.0001** |
|  |  | Related vs unrelated virgin queen | 1 | 104 | 0.48 | 0.49 |
|  | Time Point |  | 1 | 104 | 0.67 | 0.41 |
|  | Treatment*Time Point |  | 2 | 104 | 1.85 | 0.16 |
|  |  |  |  |  |  |  |
|  | **Random effects** |  | Experiment replicate | Trial | Residual |  |
|  |  |  | 4.86% | 0.34% | 94.79% |  |
| PC1 | Treatment |  | 2 | 104 | 24.59 | **<.0001** |
|  |  | Hayes vs related virgin queen | 1 | 104 | 34.91 | **<.0001** |
|  |  | Hayes vs unrelated virgin queen | 1 | 104 | 38.77 | **<.0001** |
|  |  | Related vs unrelated virgin queen | 1 | 104 | 0.10 | 0.75 |
|  | Time Point |  | 1 | 104 | 0.21 | 0.65 |
|  | Treatment*Time Point |  | 2 | 104 | 1.36 | 0.26 |
|  |  |  |  |  |  |  |
|  | **Random effects** |  | Experiment replicate | Trial | Residual |  |
|  |  |  | 4.59% | 2.00% | 93.40% |  |
| VCL | Treatment |  | 2 | 104 | 22.56 | **<.0001** |
|  |  | Hayes vs related virgin queen | 1 | 104 | 32.61 | **<.0001** |
|  |  | Hayes vs unrelated virgin queen | 1 | 104 | 35.03 | **<.0001** |
|  |  | Related vs unrelated virgin queen | 1 | 104 | 0.04 | 0.83 |
|  | Time Point |  | 1 | 104 | 0.02 | 0.87 |
|  | Treatment*Time Point |  | 2 | 104 | 1.61 | 0.20 |
|  |  |  |  |  |  |  |
|  | **Random effects** |  | Experiment replicate | Trial | Residual |  |
|  |  |  | 1.38% | 4.12% | 94.50% |  |
| VAP | Treatment |  | 2 | 104 | 23.45 | **<.0001** |
|  |  | Hayes vs related virgin queen | 1 | 104 | 32.74 | **<.0001** |
|  |  | Hayes vs unrelated virgin queen | 1 | 104 | 37.45 | **<.0001** |
|  |  | Related vs unrelated virgin queen | 1 | 104 | 0.16 | 0.69 |
|  | Time Point |  | 1 | 104 | 0.32 | 0.57 |
|  | Treatment*Time Point |  | 2 | 104 | 1.20 | 0.30 |
|  |  |  |  |  |  |  |
|  | **Random effects** |  | Experiment replicate | Trial | Residual |  |
|  |  |  | 2.67% | 2.96% | 94.37% |  |
| VSL | Treatment |  | 2 | 104 | 26.04 | **<.0001** |
|  |  | Hayes vs related virgin queen | 1 | 104 | 36.90 | **<.0001** |
|  |  | Hayes vs unrelated virgin queen | 1 | 104 | 41.11 | **<.0001** |
|  |  | Related vs unrelated virgin queen | 1 | 104 | 0.11 | 0.74 |
|  | Time Point |  | 1 | 104 | 0.40 | 0.53 |
|  | Treatment*Time Point |  | 2 | 104 | 1.21 | 0.30 |
|  |  |  |  |  |  |  |
|  | **Random effects** |  | Experiment replicate | Trial | Residual |  |
|  |  |  | 10.98% | 0.07% | 88.95% |  |
| LIN | Treatment |  | 2 | 104 | 11.68 | **<.0001** |
|  |  | Hayes vs related virgin queen | 1 | 104 | 18.00 | **<.0001** |
|  |  | Hayes vs unrelated virgin queen | 1 | 104 | 17.02 | **<.0001** |
|  |  | Related vs unrelated virgin queen | 1 | 104 | 0.01 | 0.91 |
|  | Time Point |  | 1 | 104 | 0.32 | 0.57 |
|  | Treatment*Time Point |  | 2 | 104 | 0.17 | 0.84 |
|  |  |  |  |  |  |  |
|  | **Random effects** |  | Experiment replicate | Trial | Residual |  |
|  |  |  | 11.23% | 0.00% | 88.77% |  |

**Table S6** Results of linear mixed-effects models fitted by restricted maximum likelihood in the experiments testing sperm motility in a series of queen body secretions. DF = degrees of freedom; DFDen = denominator degrees of freedom. Significant results are presented in bold.

| **Dependent variable** | **Fixed effects** | **DF** | **DFDen** | **F Ratio** | **p-value** |
| --- | --- | --- | --- | --- | --- |
| Proportion of motile sperm | Treatment | 3 | 112 | 13.01 | **<.0001** |
|  | Time Point | 1 | 112 | 3.59 | 0.06 |
|  | Treatment*Time Point | 3 | 112 | 0.56 | 0.64 |
|  |  |  |  |  |  |
|  | **Random effects** | Experiment replicate | Trial | Residual |  |
|  |  | 0.03% | 8.93% | 91.04% |  |
| PC1 | Treatment | 3 | 112 | 7.87 | **<.0001** |
|  | Time Point | 1 | 112 | 2.37 | 0.12 |
|  | Treatment*Time Point | 3 | 112 | 0.64 | 0.59 |
|  |  |  |  |  |  |
|  | **Random effects** | Experiment replicate | Trial | Residual |  |
|  |  | 0.82% | 0.00% | 99.18% |  |
| VCL | Treatment | 3 | 112 | 9.38 | **<.0001** |
|  | Time Point | 1 | 112 | 3.61 | 0.06 |
|  | Treatment*Time Point | 3 | 112 | 0.39 | 0.76 |
|  |  |  |  |  |  |
|  | **Random effects** | Experiment replicate | Trial | Residual |  |
|  |  | 1.11% | 0.00% | 98.89% |  |
| VAP | Treatment | 3 | 112 | 7.46 | **<.0001** |
|  | Time Point | 1 | 112 | 1.81 | 0.18 |
|  | Treatment*Time Point | 3 | 112 | 0.69 | 0.55 |
|  |  |  |  |  |  |
|  | **Random effects** | Experiment replicate | Trial | Residual |  |
|  |  | 0.51% | 0.00% | 99.49% |  |
| VSL | Treatment | 3 | 112 | 6.31 | **0.0005** |
|  | Time Point | 1 | 112 | 1.67 | 0.20 |
|  | Treatment*Time Point | 3 | 112 | 0.83 | 0.48 |
|  |  |  |  |  |  |
|  | **Random effects** | Experiment replicate | Trial | Residual |  |
|  |  | 0.59% | 0.00% | 99.41% |  |
| LIN | Treatment | 3 | 112 | 7.55 | **0.0001** |
|  | Time Point | 1 | 112 | 2.86 | 0.09 |
|  | Treatment*Time Point | 3 | 112 | 1.48 | 0.22 |
|  |  |  |  |  |  |
|  | **Random effects** | Experiment replicate | Trial | Residual |  |
|  |  | 0.00% | 8.13% | 91.87% |  |

**References**

[1] David G, Serres C, Jouannet P. 1981 Kinematics of human spermatozoa. *Gamete Res.* **4**, 83-95. (doi:10.1002/mrd.1120040202).

[2] Katz DF, Davis R. 1987 Automatic analysis of human sperm motion. *J. Androl.* **8**, 170-181. (doi:10.1002/j.1939-4640.1987.tb02428.x).

[3] Mortimer D. 1990 Objective analysis of sperm motility and kinematics. *Handbook of laboratory diagnosis and treatment of infertility. Boca Raton: CRC*, 97-133.

[4] Simpson JL, Humphries S, Evans JP, Simmons LW, Fitzpatrick JL. 2014 Relationships between sperm length and speed differ among three internally and three externally fertilizing species. *Evolution* **68**, 92-104. (doi:10.1111/evo.12199).

[5] Wilson-Leedy JG, Ingermann RL. 2007 Development of a novel CASA system based on open source software for characterization of zebrafish sperm motility parameters. *Theriogenology* **67**, 661-672. (doi:10.1016/j.theriogenology.2006.10.003).

[6] Werner M, Simmons LW. 2008 Insect sperm motility. *Biol. Rev.* **83**, 191-208. (doi:10.1111/j.1469-185X.2008.00039.x).

[7] Pearcy M, Delescaille N, Lybaert P, Aron S. 2014 Team swimming in ant spermatozoa. *Biol. Lett.* **10**, 20140308. (doi:10.1098/rsbl.2014.0308).

[8] den Boer SP, Baer B, Boomsma JJ. 2010 Seminal fluid mediates ejaculate competition in social insects. *Science* **327**, 1506-1509. (doi:10.1126/science.1184709).

[9] Nakagawa S, Schielzeth H. 2010 Repeatability for Gaussian and non-Gaussian data: a practical guide for biologists. *Biol. Rev. Camb. Philos. Soc.* **85**, 935-956. (doi:10.1111/j.1469-185X.2010.00141.x).

[10] R development core team. 2014 R: A language and environment for statistical computing. R Foundation for Statistical Computing, Vienna, Austria. (ISBN 3-900051-07-0).

[11] Dávila F, Chérasse S, Boomsma JJ, Aron S. 2015 Ant sperm storage organs do not have phenoloxidase constitutive immune activity. *J. Insect Physiol.* **78**, 9-14. (doi:10.1016/j.jinsphys.2015.04.005).
